# Analysis of Different Parameters Affecting Diffusion, Propagation and Survival of Staphylophages in Bacterial Biofilms

**DOI:** 10.3389/fmicb.2018.02348

**Published:** 2018-09-28

**Authors:** Silvia González, Lucía Fernández, Diana Gutiérrez, Ana Belén Campelo, Ana Rodríguez, Pilar García

**Affiliations:** Instituto de Productos Lácteos de Asturias (IPLA-CSIC), Villaviciosa, Spain

**Keywords:** *Staphylococcus aureus*, biofilms, bacteriophages, phage diffusion, phage propagation

## Abstract

The elimination of bacterial biofilms remains a major challenge due to their recalcitrant nature. Bacteriophages, viruses that infect bacteria, have been gaining increasing attention as biofilm control agents. However, the development of a successful phage-based strategy requires in-depth analysis of different parameters. It is particularly important to determine the ability of a given phage to diffuse, propagate and remain viable within the complex biofilm structure. Here, we examine some of these properties for two staphylophages, vB_SauM_phiIPLA-RODI and vB_SepM_phiIPLA-C1C. Both *Staphylococcus aureus* and *Staphylococcus epidermidis* are important opportunistic pathogens that readily form biofilms on a wide array of biotic and abiotic surfaces. Our results confirmed that both phages could penetrate through biofilms formed by several bacterial strains with varying degrees of susceptibility to the viruses and biofilm-forming abilities. However, phage penetration differed depending on the specific bacterium or combination of bacteria. The data presented here suggest that the factors determining the diffusion rate of phages in biofilms include the amount of attached biomass, susceptibility of the strain, initial phage titer, phage entrapment in the extracellular matrix, and phage inactivation. This information will help to further characterize phage-bacteria interactions within biofilm communities and will be valuable for the development of antistaphylococcal products based on these phages.

## Introduction

The relentless rise in antibiotic resistance has paved the way for the introduction of alternative antimicrobial strategies. An interesting approach is the use of bacteriophages (phages), viruses that can infect and kill bacterial cells (O’Flaherty et al., 2009; Nobrega et al., 2015; Abedon et al., 2017). Phage therapy offers some compelling advantages over conventional antibacterial strategies. Most remarkably, phages can be very target-specific, sometimes infecting only some strains of a given species, they are innocuous for humans and the environment and, last but not least, nature provides a huge reservoir of potential new variants so that new products can be under continuous development. All these interesting characteristics have led to a resurgence of bacteriophages as potential antimicrobials to be used in human and veterinary medicine or industrial settings, amongst other applications. However, there are still regulatory constraints that prevent or delay the approval and commercialization of phage-based products. In order to overcome such difficulties, the scientific community must convince the authorities about the safety and efficacy of bacteriophages (EFSA, 2012). It must be noted, however, that several phage-based products are currently on the market despite the regulatory hurdles (Fernández et al., 2018a). For example, the FDA has approved the use in food environments of products containing bacteriophages against different pathogenic bacteria such as *Listeria monocytogenes* (ListShield^TM^, developed by Intralytix, Inc., and PhageGuard Listex, developed by Micreos BV), *Salmonella enterica* (PhageGuard S, developed by Micreos BV, and SalmoFresh^TM^, developed by Intralytix, Inc.) or *Escherichia coli* (EcoShield^TM^, developed by Intralytix, Inc.).

Perhaps, one of the biggest challenges for controlling microbial pathogens is biofilm removal. Biofilms represent the most widespread lifestyle in natural and artificial environments alike. Indeed, estimates indicate that biofilms are involved in at least 65% of all bacterial infections (Potera, 1999). As a result, a good antibacterial strategy needs to be effective against biofilms. In biofilms, bacterial cells are embedded in a matrix that consists of polysaccharides, eDNA and proteins. Worryingly, microorganisms forming these structures are remarkably resistant to antibiotics and disinfectants. The resistance mechanisms inherent to the biofilm lifestyle are very diverse and include the interference of the extracellular matrix with antimicrobial agents (de la Fuente-Núñez et al., 2013). On the one hand, the matrix poses a physical barrier to the diffusion of compounds. On top of that, chemical interactions between the matrix polymers and the antimicrobials can also hinder antibacterial activity. Therefore, it is very important to demonstrate the ability of a given antimicrobial to penetrate the biofilm and reach target bacteria. Some studies have demonstrated that penetration across biofilms depends on the compound as well as the biofilm thickness and composition. For example, Anderl et al. (2000) designed an elegant protocol to quantify the penetration of antimicrobials across bacterial biofilms. In this study, biofilms of *Klebsiella pneumoniae* were formed on polycarbonate membranes placed on top of culture medium agar plates inoculated with a bacterial lawn. Subsequently, disks containing different antibiotics were placed on top of the membranes and the diameter of the inhibition zones was measured. The results demonstrated that, while ciprofloxacin could readily penetrate the biofilm, ampicillin was prevented from doing so by the accumulation of beta-lactamases in the biofilm matrix. This technique was later used to test the penetration of antibiotics through biofilms formed by *Staphylococcus aureus* and *Staphylococcus epidermidis* (Singh et al., 2010). In the case of bacteriophages, Briandet et al. (2008) showed that bacteriophage c2 could diffuse within biofilms formed by both a sensitive strain and an insensitive strain of *Lactococcus lactis* by using a fluorescent marker and further microscopic observation. On the basis of this study, it appears that the biofilm matrix does not pose a significant barrier to the diffusion of bacteriophages through the biofilm. However, while this highlights that phages can be potential antibiofilm antimicrobials, it also suggests that phages can find protection from environmental challenges within the biofilm structure. Nonetheless, further studies are still necessary to fully quantify and characterize the process of biofilm penetration by phages.

There has been a substantial amount of research aimed at the development of phage-based strategies against biofilms formed by different pathogenic bacteria, including the dangerous opportunistic pathogen *S. aureus* (Gutiérrez et al., 2016; Pires et al., 2017). This bacterium can develop biofilms on implant devices, human tissues or inert surfaces, such as those in hospital or food-industry environments (Otto, 2013). Organization into these microbial communities protects *Staphylococcus* cells from antibiotics and disinfectants, thereby hampering control of staphylococcal contamination. In that sense, bacteriophages represent an interesting antibacterial strategy. Indeed, several studies have shown that staphylophages alone or in combination with other compounds can be effective for biofilm removal (Son et al., 2010; Kelly et al., 2012). Quite recently, Gutiérrez et al. (2015) isolated two virulent staphylophages, vB_SauM_ phiIPLA-RODI (phiIPLA-RODI) and vB_SepM_ phiIPLA-C1C (phiIPLA-C1C) that exhibited antibiofilm activity against both single-species and mixed-species biofilms formed by *S. aureus* (Gutiérrez et al., 2015; González et al., 2017). Here, we set out to determine the ability of the aforementioned phages to penetrate biofilms formed by different species and strains, both sensitive and insensitive to the viruses. Additionally, we assessed whether phage diffusion was affected by formation of mixed-species biofilms. Another objective was to test the ability of the phage to remain active in the biofilm matrix of different bacteria. In order to carry out these analyses, we developed a method based on plates with transwell inserts, generally used for forming eukaryotic cells monolayers, to pre-form a bacterial biofilm and then test the behavior of phage particles following their diffusion across this structure. Overall, our findings show that staphylococcal phages can indeed penetrate and propagate in biofilms. However, the success of these processes depends on the phage dose, biofilm composition and thickness, susceptibility of the strains forming the biofilm, and phage inactivation. These results will be helpful for the development of novel phage-based antibiofilm products, and provide further insight into phage-bacteria interactions within attached microbial communities.

## Materials and Methods

### Bacterial Strains, Bacteriophages and Culture Conditions

The bacterial strains used in this study are listed in **Table 1**. Routine growth of *S. aureus* and *S. epidermidis* cultures was performed at 37°C on Baird-Parker agar plates (AppliChem, Germany) or in TSB (Tryptic Soy Broth, Scharlau, Barcelona, Spain) with shaking in an orbital incubator at 250 rpm. When necessary, TSB was supplemented with glucose at a final concentration of 0.25% (TSBG) or with agar at a final concentration of 0.7% (soft-TSA) or 2% (TSA). *L. plantarum* 55-1 was grown on MRS (Scharlab S.L., Spain) broth or agar at 32°C. Bacteriophages phiIPLA-RODI and phiIPLA-C1C were, respectively, propagated on *S. aureus* IPLA 1 and *S. epidermidis* F12 as previously described (Gutiérrez et al., 2010).

**Table 1:** Bacterial strains and bacteriophages used in this study.

Bacterial strains/bacteriophages	Source^∗^	Reference
**Bacterial strains**		
***Staphylococcus aureus***		
IPLA1	Dairy industry surface	Gutiérrez et al. (2012)
IPLA15 (Sa IPLA15)	Meat industry surface	Gutiérrez et al. (2012)
IPLA16 (Sa IPLA16)	Meat industry surface	Gutiérrez et al. (2012)
RN450 (Sa RN450)	Derivative of strain NCTC8325	Novick (1967)
ISP479r (Sa ISP479r)	Derivative of strain NCTC8325	Toledo-Arana et al. (2005)
V329 (Sa V329)	Bovine subclinical mastitis	Cucarella et al. (2001)
***Staphylococcus epidermidis***		
F12 (Se F12)	Milk from woman with mastitis	Delgado et al. (2009)
Z2LDC14 (Se Z2LDC14)	Milk from woman with mastitis	Delgado et al. (2009)
DG2ñ (Se DG2ñ)	Milk from woman with mastitis	Delgado et al. (2009)
***Lactobacillus plantarum***		
55-1 (Lp 55-1)	Natural fermentation of olives	Ruiz-Barba et al. (1994)
**Bacteriophages**		
vB_SauM_ phiIPLA-RODI	Isolated from STP^∗^	Gutiérrez et al. (2015)
vB_SepM_ phiIPLA-C1C	Isolated from STP^∗^	Gutiérrez et al. (2015)


*^∗^STP, sewage treatment plant.*

### Biofilm Formation Assays

To assess the biofilm-forming ability of the different strains used in the study, 450 μl from cell suspensions containing 10^6^ CFU/ml in TSBG were inoculated into the inserts of 24-well Transwell^®^ plates containing polycarbonate membranes (Corning, NY, United States). In the case of biofilms formed by two strains, we mixed the corresponding cells suspensions 1:1 prior to inoculating the inserts. Biofilms were allowed to develop for 24 h at 37°C and, following incubation, the attached biomass was stained with 0.1% crystal violet according to the protocol described previously (Gutiérrez et al., 2014). However, the volume used for washing, staining and distaining steps was 400 μl instead of 200 μl. Absorbance at 595 nm (A_595_) was then quantified by using a Bio-Rad Benchmark Plus Microplate Spectrophotometer (Bio-Rad Laboratories, Hercules, CA, United States). The obtained values represent the crystal violet retained in the samples after the washing steps, which is, in turn, an indirect measurement of total attached biomass (cells and matrix). According to their A_595_ values, strains were categorized as strong (A_595_ ≥ 2), intermediate (1 < A_595_ < 2), and weak (A_595_ ≤ 1) biofilm formers.

### Confocal Laser Scanning Microscopy (CLSM)

Each well of a 2-well μ-slide with a glass bottom (ibidi, United States) was inoculated with 1 ml of a cell suspension containing 10^6^ cfu/ml in TSBG. Biofilms were then allowed to form for 24 h at 37°C. After biofilm development, the planktonic phase was removed and wells were washed with PBS prior to staining all cells (live and dead) with SYTO 9 from the LIVE/DEAD^®^ BacLight^TM^ Kit (Invitrogen AG, Basel, Switzerland) as indicated by the manufacturer. The biofilm samples were then observed under a DMi8 confocal laser scanning microscope (Leica Microsystems) using a 63× oil objective.

### Determination of the Minimum Inhibitory Multiplicity of Infection (MIMOI)

The susceptibility of the different strains to phages phiIPLA-RODI and phiIPLA-C1C was assessed by a modification of the broth microdilution assay as described elsewhere (Fernández et al., 2017). The MIMOI was considered to be the lowest multiplicity of infection or MOI (number of phage particles per bacterial cell) at which no bacterial growth was observed with the naked eye, as indicated in the Clinical Laboratory Standards Institute (2015), following 24 h of incubation at 37°C.

### Bacteriophage Diffusion Assays

**FIGURE 1:**
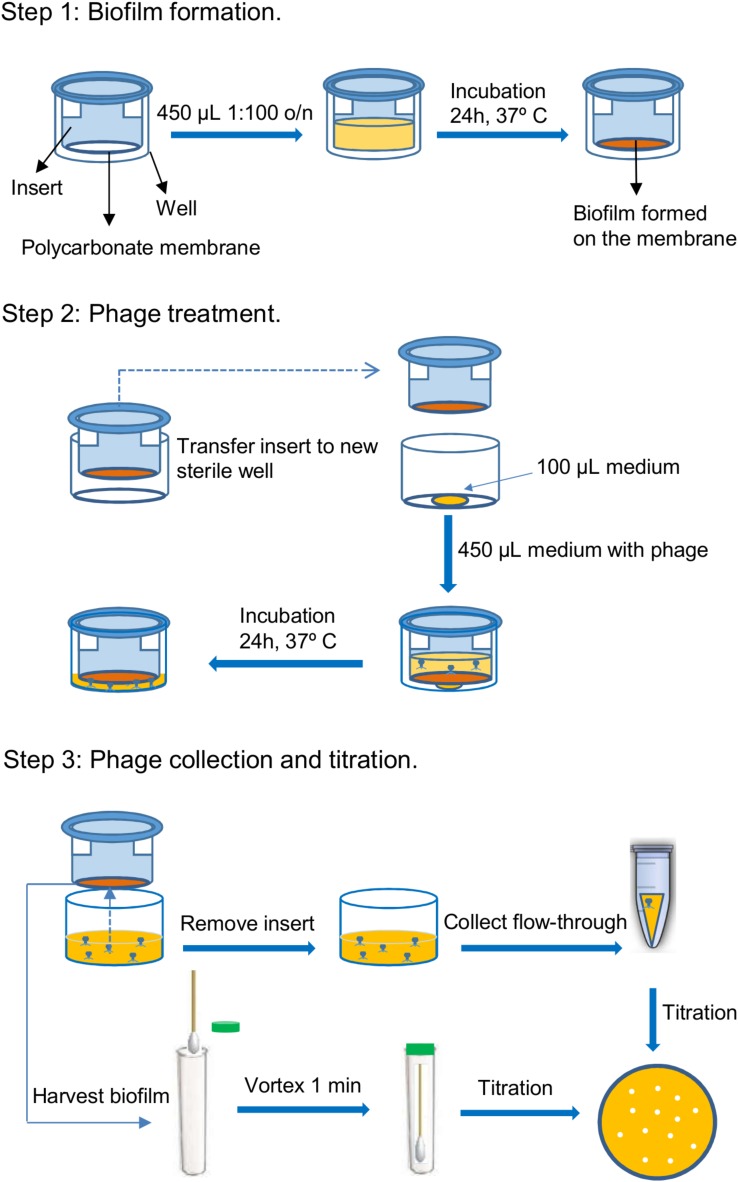
Schematic representation of the steps involved in the protocol for testing the penetration of bacteriophages through bacterial biofilms. These steps are the following: 1. Biofilm formation, 2. Phage treatment, and 3. Phage collection and titration.

A schematic representation of the protocol used to assess phage penetration across bacterial biofilms is depicted in **Figure 1**. In a first step, biofilms were pre-formed on polycarbonate membranes with 0.4 μm pore size placed inside the inserts of 24-well Transwell^®^ plates (Corning, NY, United States). To do that, 450 μl from a cell suspension containing 10^6^ CFU/ml in fresh TSBG were inoculated into each insert and incubated for 24 h at 37°C. Control wells were inoculated with 450 μl of TSBG medium. After incubation, the inserts were placed into new sterile wells containing a 100-μl drop of TSB, as this was found to facilitate movement of the liquid across the membrane. Next, 450 μl of phage suspensions containing 10^6^ or 10^9^ PFU/ml in TSB were carefully poured on top of the pre-formed biofilms. The plates were then further incubated for 24 h at 37°C, during which time the liquid moved across the polycarbonate membrane from the insert to the bottom chamber. The following day, the phage titer in the flow-through and the biofilm were determined by the double-layer assay (Gutiérrez et al., 2010). The biofilm was washed with PBS and then gently scraped from the membrane with a sterile cotton swab and then resuspended in 5 ml of SM buffer (20 mg/liter Tris–HCl, 10 mg/liter MgSO_4_, 10 mg/liter CaCl_2_, 100 mg/liter NaCl, pH 7.5) by vortexing the sample for 1 min. This suspension was then titrated to determine the number of phage particles trapped in the extracellular matrix. Then, the insert was removed and the flow-through was collected from the bottom chamber. Serial dilutions of these suspensions were used for phage titration.

Analysis of the correlation between biofilm formation or susceptibility and phage titer in the flow-through was performed by calculating indicators of these two properties. The indicator of biofilm formation ability was the average A_595_ resulting from crystal violet staining of biofilms for a given strain. Calculation of the susceptibility values was based on the results obtained in the MIMOI determination assays. The minimum susceptibility, with a value of 0, was assigned to the non-susceptible strains. In turn, the most susceptible strains for each phage were assigned a susceptibility value of 5. Susceptibility of the other strains was calculated by subtracting the change in logarithmic units between their MIMOIs and that of the most susceptible strain (strain MS):

Susceptibility of strain A=5-log [MIMOI (strain A)/MIMOI(strain MS)]

Finally, a third indicator combining the two previous properties was calculated as the quotient susceptibility/biofilm formation. The results obtained in these three calculations for the different strains were then plotted against log_10_ (PFU/ml) in which PFU/ml corresponds to the phage titer in the flow-through. Subsequently, the values in the resulting chart were fitted to a trend line and the coefficient of determination (R^2^) was calculated to determine the goodness of fit.

### Phage Inactivation Assay

Different bacterial strains were grown overnight at 37°C in TSB or TSBG without shaking. Control samples with medium alone were also included. The following day, supernatants were collected and filtered after centrifugation at 13,200 rpm for 3 min. 900 μl aliquots from each sample were taken and mixed with 100 μl of a phage suspension to obtain a final concentration of 10^6^ PFU/ml. All samples were subsequently incubated at 37°C for 3 h. After incubation, the phage titer was determined as described above. The pH of the different supernatants was determined by using pH-indicator strips (Merck, Darmstadt, Germany).

### Statistical Analyses

All phage penetration experiments were performed with at least four technical repeats corresponding to two independent biological replicates. Biofilm formation, MIMOI determination and phage inactivation assays were performed with three independent biological replicates. Data was then analyzed with a two-tailed Student’s *t*-test and significance was set at a *P*-value threshold of 0.05.

## Results

### Biofilm Formation on Polycarbonate Membranes and Phage Susceptibility of Different Bacterial Strains

The aim of this study was to determine the impact of certain factors on the success of bacteriophage diffusion across bacterial biofilms. Amongst other factors, we wanted to examine the effect of biofilm-forming ability and phage susceptibility. To do that, it was necessary to ensure that the strains selected for the study represent varying degrees of these two characteristics prior to performing the biofilm penetration experiments. First, several strains of *S. aureus* and *S. epidermidis* were chosen based on previous data (Gutiérrez et al., 2014, 2015), as well as *Lactobacillus plantarum* 55-1. This bacterium can form mixed biofilms with *S. aureus* and is not susceptible to phage phiIPLA-RODI (González et al., 2017).

The assessment of biofilm formation was carried out on the polycarbonate membranes of transwell plates that would be subsequently used for phage penetration tests. Total biomass (cells plus matrix) attached to the membranes following 24 h of incubation at 37°C was quantified by crystal violet staining and subsequent measurement of absorbance at 595 nm (A_595_). The results of these assays revealed that these bacterial strains display a wide range of biofilm-forming abilities. For the sake of this study, strains were classified as weak (A_595_ ≤ 1), intermediate (1 < A_595_< 2) and strong (A_595_ ≥ 2) biofilm formers (**Table 2** and **Supplementary Figure S1A**). According to this criterion, strains *S. aureus* V329 (Sa V329) and *S. aureus* ISP479r (Sa ISP479r) were considered strong biofilm formers, whereas *L. plantarum* 55-1 (Lp 55-1) and *S. aureus* RN450 (Sa RN450) were weak biofilm formers (**Table 2** and **Supplementary Figure S1A**). All other strains displayed an intermediate ability to develop biofilms on polycarbonate membranes (**Table 2** and **Supplementary Figure S1A**). These strains included *S. aureus* IPLA15 (Sa IPLA15), *S. aureus* IPLA16 (Sa IPLA16), *S. epidermidis* F12 (Se F12), *S. epidermidis* Z2LDC14 (Se Z2LDC14) and *S. epidermidis* DG2ñ (Se DG2ñ). Besides being a strong biofilm former, strain Sa V329 is also interesting as it is the only *S. aureus* strain included here whose biofilm matrix is principally composed of proteins instead of polysaccharide (Gutiérrez et al., 2014).

**Table 2:** MIMOI values for phages phiIPLA-RODI and phiIPLA-C1C and biofilm-forming ability of the different bacterial strains used in this study.

Bacterial strain	phiIPLA-RODI	phiIPLA-C1C	Biofilm formation^1^
Sa IPLA15	100	NS	1.18 ± 0.24 (intermediate)
Sa IPLA16	0.01	1	1.48 ± 0.39 (intermediate)
Sa RN450	100	NS	0.72 ± 0.04 (weak)
Sa ISP479r	0.1	NS	2.04 ± 0.15 (strong)
Sa V329	10	NS	3.37 ± 0.82 (strong)
Se F12	NS	1	1.36 ± 0.38 (intermediate)
Se Z2LDC14	NS	0.1	1.86 ± 0.07 (intermediate)
Se DG2ñ	NS	100	1.66 ± 0.48 (intermediate)
Lp 55-1	NS	NS	0.64 ± 0.15 (weak)


*^∗^NS, not susceptible; ^1^A_595_ was determined after crystal violet staining of 24-h biofilms formed on polycarbonate membranes at 37°C. Based on these values, strains were classified into strong (A_595_ > 2), intermediate (1 < A_595_ < 2) and weak (A_595_ < 1) biofilm formers.*

Visual inspection of the polycarbonate membranes after incubation of the bacterial cultures showed the development of biofilm all over the membrane, from edge to edge. However, this does not necessarily mean that biofilm coverage and architecture is homogeneous at the microscopic level. For this reason, we examined the structure of biofilms formed by the different strains on glass-bottomed slides by CLSM. The thickness of the observed biofilms showed a relatively good correlation (0.6105) with the crystal violet data from biofilms grown on polycarbonate membranes (**Supplementary Figure S1B**). Regarding surface coverage, most strains seem to have developed biofilms that covered the slide bottom rather homogeneously, with the exception of Sa IPLA15 and Se F12 (**Supplementary Figures S2**, **S3**). There were also differences between strains regarding the complexity of biofilm architecture (**Supplementary Figures S2**, **S3**). For instance, the two strong biofilm-forming strains, namely ISP479r and V329, showed notable differences, with the latter (V329) exhibiting a much more complex 3D structure (**Supplementary Figure S2**).

Besides biofilm-forming ability, susceptibility of the different strains to phages phiIPLA-RODI and phiIPLA-C1C was also assessed by determining their MIMOI values (**Table 2**). As is the case with the minimum inhibitory concentrations (MICs) of antibiotics, a higher MIMOI represents a lesser susceptibility to the phage. Based on this criterion, the most susceptible strains to phiIPLA-RODI were Sa IPLA 16, Sa ISP479r and Sa V329, while the other two *S. aureus* strains, Sa IPLA15 and Sa RN450, required a higher MOI to inhibit visible growth (**Table 2**). Finally, all the *S. epidermidis* strains and Lp 55-1 were insensitive to phiIPLA-RODI. Regarding phage phiIPLA-C1C, susceptible strains included Se Z2LDC14, Se F12, Sa IPLA16 and Se DG2ñ, while *L. plantarum* and most *S. aureus* strains (Sa IPLA15, Sa ISP479r, Sa RN450, and Sa V329) were insensitive to this phage (**Table 2**).

### Diffusion of phiIPLA-RODI and phiIPLA-C1C Through Bacterial Biofilms

Once established that the selected strains displayed different degrees of biofilm-forming ability and susceptibility to the phages, penetration of phiIPLA-RODI and phiIPLA-C1C across bacterial biofilms was examined following the protocol depicted in **Figure 1**. To do that, 24-h biofilms were formed on the polycarbonate membranes of transwell plates and subsequently treated with different phage suspensions (**Figure 1**). Following incubation for another 24 h at 37°C, the liquid from the insert had moved across the biofilm and the membrane to the bottom chamber, and phage titer in the flow-through was determined to assess phage penetration. The results of biofilm-containing wells were compared to those of control wells (without biofilm) to correct for the possibility of bacteriophage particles being retained in the polycarbonate membrane.

**FIGURE 2:**
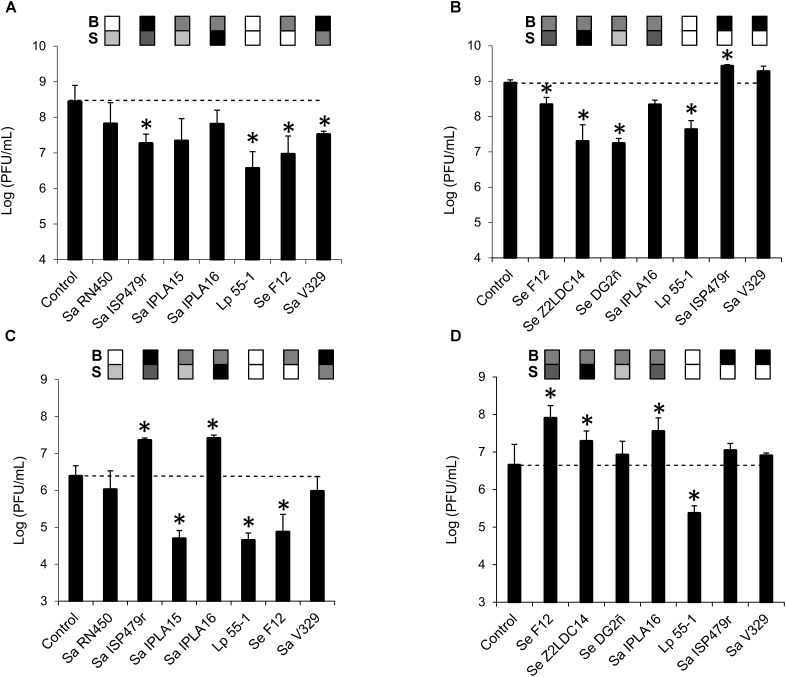
Diffusion of bacteriophages phiIPLA-RODI and phiIPLA-C1C through bacterial biofilms formed by different strains. Biofilms were pre-formed on the membranes of transwell plates for 24 h at 37°C and subsequently treated with 10^9^ **(A,B)** or 10^6^ **(C,D)** PFU/ml of bacteriophages phiIPLA-RODI **(A,C)** or phiIPLA-C1C **(B,D)**. Phage particles in the flow-through were titrated after incubation at 37°C for 24 h. The values represent the average and standard deviation of at least three replicates. Differences compared to the control (membrane without biofilm) with *P*-values < 0.05 were considered significant (^∗^). Sa, *S. aureus*; Se, *S. epidermidis*; and Lp, *L. plantarum*. The grid on top of each bar represents the biofilm strength (B) and susceptibility (S) of each strain in a color-coded manner. For biofilm strength, black, gray, and white represent strong, intermediate and weak biofilm formation. Susceptibility is represented in a scale that goes from white (non-susceptible strains) to black (most susceptible strain for a given phage).

In a first experiment, penetration was tested by treating pre-formed 24-h-old biofilms with phage suspensions containing a high titer (∼10^9^ PFU/ml) of phages phiIPLA-RODI or phiIPLA-C1C. Phage titer in the flow-through was determined and compared to that of control non-inoculated wells. In the bacteria-inoculated membranes, phage penetration occurred in all samples but the titer in the flow-through varied depending on the specific strain. Thus, the titer of phiIPLA-RODI in the flow-through was significantly lower for strains Sa ISP479r, Lp 55-1, Se F12, and Sa V329. In contrast, the phage titer obtained for biofilms formed by Sa RN450, Sa IPLA 15, and Sa IPLA 16 was not significantly different from that of the control (**Figure 2A**). These three *S. aureus* strains were poor or intermediate biofilm formers (**Table 2**). However, even though Se F12 and Lp 55-1 did not form robust biofilms either, the phage titer in the flow-through was approximately 2 log units lower than that of the control. Interestingly, Se F12 and Lp 55-1 share their lack of susceptibility to phiIPLA-RODI. In the case of phiIPLA-C1C, a decrease in phage titer was observed for the biofilms formed by Se F12, Sa IPLA16, Se Z2LDC14, Se DG2ñ, and Lp 55-1. Indeed, the latter three strains showed reductions in the phage titer of up to 2 log units, despite the fact that none of them are strong biofilm formers and that two of them (Se Z2LDC14 and DG2ñ) are susceptible to the phage. Conversely, no decrease was observed for Sa V329 or Sa ISP479r (**Figure 2B**).

The assays performed with a high phage titer revealed different penetration rates for the phages depending on the bacterial strain forming the biofilm. However, they did not give any indication of whether propagation was taking place during this process. Perhaps this was due to the fact that the phage concentrations used were near the maximum propagation rates observed for these phages, thereby masking this phenomenon. Taking this into consideration, the assay was repeated by treating the biofilms with a lower phage titer (∼10^6^ PFU/ml). The results obtained here were quite different from those in the previous experiment (**Figures 2C,D**). Thus, while the phage titer remained unchanged or decreased for some strains, there were examples in which a significant increase in phage titer in the flow-through was detected. This was the case, for instance, of strains Sa ISP479r and Sa IPLA 16 for phage phiIPLA-RODI (**Figure 2C**) or Se F12, Se Z2LDC14, and Sa IPLA 16 for phiIPLA-C1C (**Figure 2D**). As would be expected, these were the most susceptible strains to each phage. Instead of phage propagation, a decrease in phage titer was observed for the other strains. Notably, the phage titer of the insensitive bacterium Lp 55-1 exhibited a decrease of almost 2 log units for both viruses. Additionally, there were significant reductions for biofilms formed by Sa IPLA15 and Se F12 in the case of phiIPLA-RODI. This is interesting because neither of these two strains is a very good biofilm former and Sa IPLA15 shows susceptibility, albeit not very high, to this phage. Finally, no significant changes in phage titer were observed for strains Sa RN450 and Sa V329 in the case of phiIPLA-RODI, or strains Se DG2ñ, Sa ISP479r, and Sa V329 for phage phiIPLA-C1C.

In an attempt to examine the influence of biofilm-forming ability and phage susceptibility on phage diffusion across biofilms, these values were plotted against the phage titers quantified in the flow-through to detect potential trends (**Supplementary Figures S4**, **S5**). The indicators of biofilm-forming capacity and susceptibility were estimated as previously described (see Materials and Methods). The results of linear regression analysis obtained for a high concentration (10^9^ PFU/ml) of phage phiIPLA-RODI indicated that the most acceptable model for predicting the phage titer in the flow-through of different biofilms was the one that considered both susceptibility and biofilm formation (*R*^2^ = 0.554), whereas susceptibility and biofilm formation alone showed fairly low fitting coefficients (*R*^2^ < 0.5) (**Supplementary Figure S4**). We observed that there was a positive correlation between both susceptibility and susceptibility/biofilm formation and phage titer in the flow-through as indicated by the fact that both trend lines had a positive slope (**Supplementary Figure S4**). When treatment was carried out with a low phage concentration (10^6^ PFU/ml) of phiIPLA-RODI, the best predictive model was obtained when plotting phage titer in the flow-through against susceptibility (*R*^2^ = 0.872) (**Supplementary Figure S4**), which indicated that there was a positive correlation between the two variables. Analysis of the data obtained for phage phiIPLA-C1C, however, gave very different results. Thus, when treatment of bacterial biofilms was performed with a high phage concentration (10^9^ PFU/ml) the most acceptable model was obtained by plotting phage titer against biofilm-forming ability (*R*^2^ = 0.3417), although fitting was still very low (*R*^2^ < 0.5) (**Supplementary Figure S3**). In the case of samples treated with a low concentration (10^6^ PFU/ml) of phiIPLA-C1C, the best-fitting model was obtained for phage susceptibility/biofilm formation (*R*^2^ = 0.5225), which was slightly better than the model that represented phage titer against susceptibility (*R*^2^ = 0.4566). The correlation between susceptibility or susceptibility/biofilm formation and phage titer in the flow-through was positive. Overall, it seems that penetration of phage phiIPLA-RODI through biofilms is positively correlated with susceptibility of the bacterial strain after treatment with both high and low phage concentrations (**Supplementary Figure S4**). Moreover, at high phage concentrations the effect of susceptibility is further modulated by the biofilm-forming ability of the strain (**Supplementary Figure S4**). In contrast, penetration of phiIPLA-C1C does not appear to be highly correlated to biofilm formation or susceptibility of the strains involved, with perhaps additional undetermined factors playing a role in phage diffusion (**Supplementary Figure S5**).

### Penetration of phiIPLA-RODI Through Mixed-Species Biofilms

**FIGURE 3:**
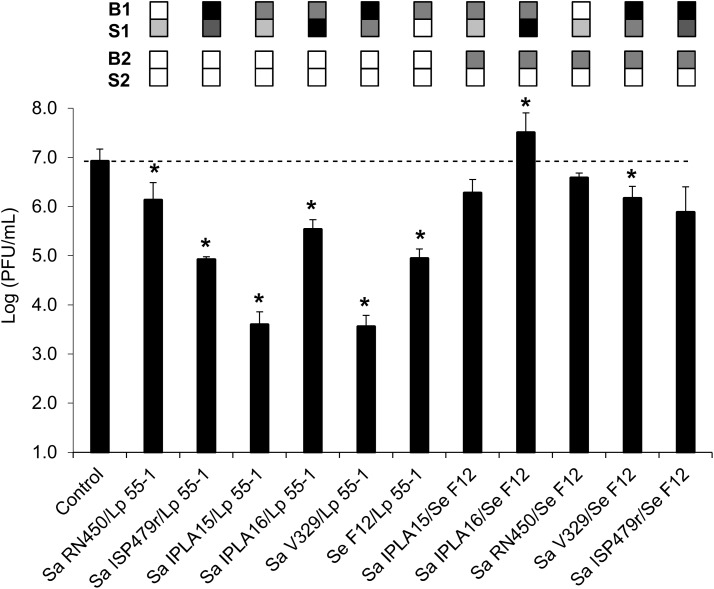
Diffusion of phage phiIPLA-RODI through mixed-species biofilms. The ability of phage suspensions containing 10^6^ PFU/ml to diffuse across biofilms formed by a combination of two strains (strain 1/strain 2) was examined. The changes observed in mixed-strain biofilms were compared to those observed when the same bacteria were forming single-strain biofilms. Differences compared to the control (membrane without biofilm) with *P*-values < 0.05 were considered significant (^∗^). Sa, *S. aureus*; Se, *S. epidermidis*; and Lp, *L. plantarum*. The grid on top of each bar represents the biofilm strength (B) and susceptibility (S) of each strain in a color-coded manner. For biofilm strength, black, gray, and white represent strong, intermediate and weak biofilm formation. Susceptibility is represented in a scale that goes from white (non-susceptible strains) to black (most susceptible strain for a given phage). For each pair forming a mixed biofilm, B1 and S1 correspond to the first strain while B2 and S2 correspond to the second strain (*L. plantarum* or *S. epidermidis*).

Further experiments were performed to analyze more in-depth the properties that affect phage penetration through bacterial biofilms. In particular, we evaluated the ability of phage phiIPLA-RODI applied at a low phage titer (∼10^6^ PFU/ml) to diffuse across mixed-species biofilms. A previous study had shown that the propagation success of phiIPLA-RODI in multispecies biofilms formed by Sa IPLA16 differed depending on the accompanying species (González et al., 2017). Precisely one of the species used in the cited study was *L. plantarum*, which showed a good ability to form mixed-species biofilms with *S. aureus*. Indeed, the difference in viable cell counts in mixed biofilms of the two bacteria were less than one logarithmic unit and microscopy analysis indicated that cells of both species were mixed throughout the biofilm. The ability of *S. epidermidis* to form mixed biofilms with *S. aureus* with similar viable cell counts of the two species had been previously shown by Gutiérrez et al. (2015). In the present study, mixed-species biofilms were formed by inoculating different combinations of *S. aureus* strains with Se F12 or Lp 55-1, as well as a mixed biofilm formed by Se F12 and Lp 55-1. The results obtained were then compared to data from control wells (without biofilms). In general, the presence of Lp 55-1 led to a decrease in the phage titer of the flow-through compared to the non-inoculated control well, regardless of the accompanying strain (**Figure 3**). However, in some cases, this reduction was significantly smaller than that observed for the Lp 55-1 monospecies biofilms (2 log units, **Figure 2C**), as was the case of *L. plantarum* combined with Sa RN450 (1 log unit, **Figure 3**). Conversely, there was an increased reduction in phage titer compared to *L. plantarum* alone (2 log units, **Figure 2C**) when Lp 55-1 formed a biofilm with Sa IPLA15 or Sa V329 (3 log units, **Figure 3**). Perhaps, the mechanisms by which there is a decrease in the phage titer in these *S. aureus* strains and that of *L. plantarum* cultures have a cumulative effect. Mixed species biofilms of Lp 55-1 with Sa IPLA16 or Se F12 showed no significant difference compared to Lp 55-1 alone (2 log units decrease compared to the control wells, **Figure 3**). Overall, it seems quite clear that Lp 55-1 has a negative impact on the viability of phage phiIPLA-RODI.

Despite the fact that Lp 55-1 and Se F12 monospecies biofilms behaved similarly, the results obtained for mixed biofilms formed by Se F12 with different *S. aureus* strains were quite different from those displayed by mixed biofilms of the same strains with *L. plantarum* (**Figure 3**). Indeed, most combinations of Se F12 with *S. aureus* led to a lesser decrease in phage titer in the flow-through than that observed for Se F12 monospecies biofilms (2 log units, **Figure 2C**). This was, for instance, the case for multispecies biofilms of Se F12 with Sa IPLA15 (no change, **Figure 3**), Sa RN450 (no change, **Figure 3**), Sa ISP479r (no change, **Figure 3**) and Sa V329 (1 log unit decrease, **Figure 3**). Moreover, development of mixed biofilms of Se F12 and Sa IPLA 16 allowed for phage propagation to occur (1 log unit increase compared to control wells, **Figure 3**). Interestingly, the combination of Sa IPLA15 with Se F12 led to a significantly lower decrease in phage titer than either bacterium alone, which is exactly the opposite to the results obtained when Sa IPLA15 was grown with Lp 55-1. Therefore, it seems that the mechanisms involved in Se F12 and Sa IPLA15 reduction in phage titer somehow counteract each other. Also, the phage titer in the flow-through obtained when treating mixed-strain biofilms formed by Sa IPLA15 and Sa ISP479r exhibited an intermediate result between those observed for the two monospecies biofilms. Overall, combination of Se F12 with any of the *S. aureus* strains seems to prevent the decrease in the phage titer observed for the *S. epidermidis* strain alone, which is in stark contrast to the results displayed by mixed biofilms involving Lp 55-1. This suggests that phage inactivation mechanisms differ between the two microorganisms.

### Titration of Phage Particles Retained in the Biofilm Matrix

**FIGURE 4:**
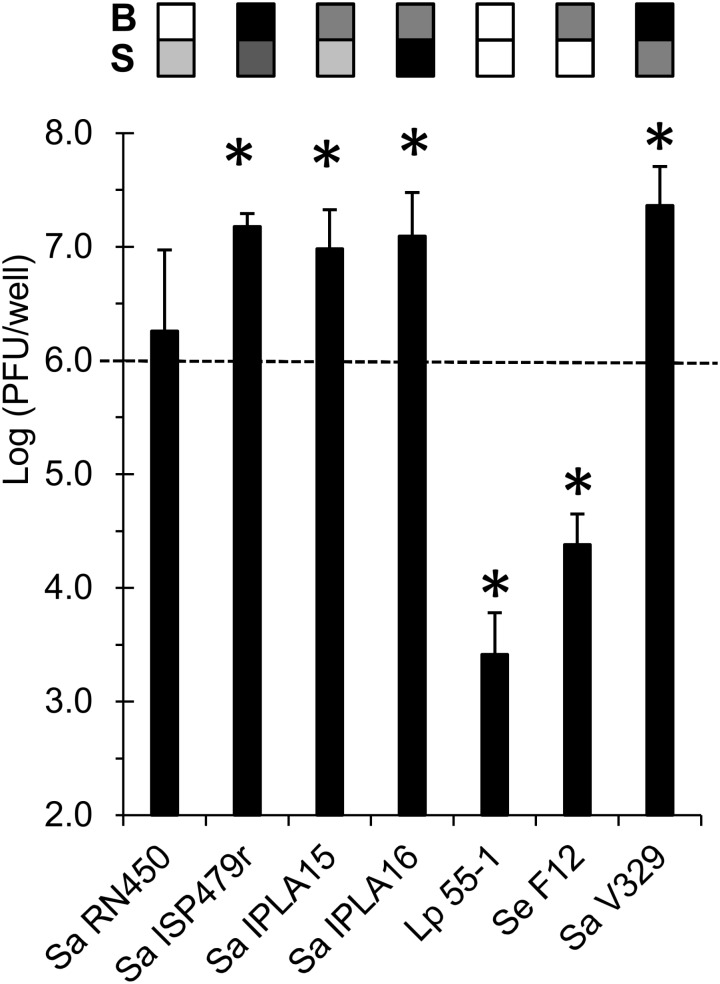
Phage titer in the extracellular matrix of biofilms formed by the different bacterial strains treated with 10^6^ PFU/ml of phage phiIPLA-RODI after incubation for 24 h at 37°C. The values represent the average and standard deviation of the PFUs per well obtained for at least three replicates. These values were compared to the inoculum (PFU/well) and *P*-values < 0.05 were considered significant (^∗^). The grid on top of each bar represents the biofilm strength (B) and susceptibility (S) of each strain in a color-coded manner. For biofilm strength, black, gray, and white represent strong, intermediate and weak biofilm formation. Susceptibility is represented in a scale that goes from white (non-susceptible strains) to black (most susceptible strain for a given phage).

One possible explanation for the reduced phage titer observed in the flow-through for some strains is the entrapment of the virus in the extracellular matrix of the biofilm. To study this possibility, we analyzed the amount of viable phage phiIPLA-RODI particles trapped in the biofilms formed by the different strains after treatment with a low phage concentration (∼10^6^ PFU/ml). These values were then compared to the number of phage particles present in the inoculum. Interestingly, the results of this experiment did not completely mirror those obtained in the analysis of the flow-through. Here, most strains showed a significant change in phage titer compared to the inoculum, with the only exception of Sa RN450 (**Figure 4**). In some cases, there was a one log unit increase in phage titer in the biofilm compared to the inoculum. More specifically, this occurred for strains Sa ISP479r, Sa IPLA15, Sa IPLA16, and Sa V329, all of which showed susceptibility to this phage. It is worth noting the fact that Sa IPLA15, which displayed a significant decrease in phage titer in the flow-through (**Figure 2C**), and Sa V329, which did not show any change in the flow-through (**Figure 2C**), exhibited evidence of phage propagation in the biofilm. Therefore, it appears that part of the phage population may be trapped within the extracellular matrix and does not diffuse easily through the biofilm. Alternatively, there may be phage inactivation during diffusion of the phage suspension across the biofilm and subsequent phage propagation in the biofilm. Regarding Sa RN450, whose susceptibility to phiIPLA-RODI is similar to that of Sa IPLA15, the results were more variable than for the other strains and, when taken together, did not give indication of a significant propagation in the biofilm. Conversely, the insensitive strains Lp 55-1 and Se F12 displayed a lower phage titer (1-2 log units) in the biofilm than in the inoculum. Therefore, phage phiIPLA-RODI titers for these two strains are low in both the biofilm matrix and the flow-through. This result suggests inactivation of a significant part of the initial phage population, although the specific mechanisms involved remain to be elucidated. Interestingly, regression analysis of the values obtained for phage entrapped in the biofilm matrix compared to the phage titer in the flow-through for the same samples did not show a high correlation unless the data point corresponding to strain Sa IPLA 15 was taken out (**Supplementary Figure S6**). Indeed, *R*^2^ was 0.445 when all strains were considered, but increased up to *R*^2^ = 0.7966 if Sa IPLA15 was taken out of the analysis.

### Inactivation of Phage phiIPLA-RODI by Bacterial Supernatants

The significant depletion in active viral particles in most biofilms formed by *L. plantarum* 55-1, either alone or as part of mixed cultures, suggested that this bacterium might somehow inactivate the phage. On the other hand, data obtained thus far suggests that strain Se F12 also inactivates phiIPLA-RODI, although perhaps through a different mechanism. To explore these hypotheses, phage inactivation by supernatants of these two strains was examined. The experiment was performed with supernatants from overnight cultures of the two bacteria grown in TSBG (medium used for biofilm formation) and TSB (medium used for phage treatment step). Our results were different depending on the culture medium used. Thus, when bacteria were grown in TSB there were no significant changes in phage survival compared to the medium control (**Table 3**). In contrast, supernatants from cultures grown in TSB supplemented with 0.25% glucose (TSBG) led to a significant loss in phage viability after incubation at 37°C for 3 h (**Table 3**). Indeed, following incubation with the supernatant from strain Lp 55-1, the phage titer of the phage suspension was below the detection level (<10). In contrast, a significant but lesser degree of inactivation (about 1 log unit) was observed for strain Se F12.

**Table 3:** Survival of phiIPLA-RODI after incubation with bacterial supernatants.

Bacterial strain	TSBG^∗^	TSB^∗^
Control	1.27 × 10^6^ ± 6.11 × 10^4^ (pH = 7)	1.46 × 10^6^ ± 4.91 × 10^5^ (pH = 7)
Lp 55-1	<10 (#) (pH = 4)	1.31 × 10^6^ ± 2.08 × 10^4^ (pH = 5)
Se F12	1.75 × 10^5^ ± 7.63 × 10^4^ (#) (pH = 5)	1.91 × 10^6^ ± 3.87 × 10^5^ (pH = 6)


*^∗^Phage titer (PFU/ml) following incubation for 3 h at 37°C of a phage suspension in medium (control) or in supernatants of overnight liquid cultures of different bacterial strains. Values represent the average and standard deviation of three replicates. Values obtained with treated samples were compared to the control and *P*-values < 0.05 were considered significant (#). The pH of the different supernatants prior to the experiment is indicated in brackets.*

Besides phage inactivation, we examined the pH of the different supernatants. When grown in TSBG, *L. plantarum* and *S. epidermidis* acidified the medium to pH values of 4 and 5, respectively. These values were exactly the same as those observed in the flow-through for Lp 55-1 and Se F12 when performing the phage-penetration experiments after the biofilm-formation step in TSBG. In contrast, the pH of cultures grown in TSB was 5 and 6 for *L. plantarum* and *S. epidermidis*, respectively. These values were exactly the same as those observed in the flow-through for Lp 55-1 and Se F12 when performing the phage-penetration experiments after the phage treatment step in TSB. Taking these results into consideration, it does not appear that lowering of the pH during phage treatment is fully responsible for the decrease in phage titer observed for biofilms of Lp 55-1 and Se F12. Perhaps, additional factors such as production of extracellular enzymes also play a role in phage inactivation.

## Discussion

Bacterial cells are most frequently organized in structured multicellular communities, biofilms, in which they can withstand environmental sources of stress that would easily kill them in the planktonic state. Indeed, one of the most challenging problems in bacterial contamination control is precisely biofilm eradication. Virulent bacteriophages are currently being considered as promising antibiofilm agents thanks to their ability to infect and lyse bacterial cells (Pires et al., 2017). However, the development of phage-based antimicrobial products aimed for biofilm removal requires careful consideration of several aspects of phage-host interactions within biofilms (Fernández et al., 2018a,b). One of the properties that must be explored is the ability of the phage to penetrate the tight-knit network of bacterial cells and matrix polymers that form these complex communities. This study describes the development of a new method that facilitates the analysis of phage diffusion as well as the potential factors affecting the ability of the virus to move across the biofilm. This technique is based on the use of transwell plates, which allow separating two chambers by a polycarbonate membrane. Coincidentally, a recent article used these plates to demonstrate that bacteriophages can move across human cell layers by transcytosis (Nguyen et al., 2017). So far, studies on the diffusion of bacteriophages through bacterial biofilms are scarce. For example, Briandet et al. (2008) analyzed diffusion of lactococcal phage c2 across biofilms formed by susceptible and non-susceptible strains by fluorescence correlation spectroscopy. This study found that the phage could diffuse across biofilms formed by the insensitive strain, *Stenotrophomonas maltophilia*, as well as a sensitive and a resistant strain of *Lactococcus lactis*. In both lactococcal strains, however, phage particles appeared to be immobile, suggesting binding to cell surface receptors. More recently, a computer simulation framework indicated that phage mobility may have an impact on phage-host interactions within the biofilm structure (Simmons et al., 2017).

One of the main target microorganisms of phage therapy research is the human pathogen *S. aureus*. Some strains of this bacterium have become resistant to a wide range of antimicrobials, including some of the last resort drugs currently used in the clinic. To make matters worse, this microbe can form biofilms on inert surfaces or human tissues and persist, thereby increasing danger of contamination as well as hindering infection treatment. Several studies have demonstrated that phages can be an option for treating staphylococcal infections, including those involving biofilms (Son et al., 2010; Kelly et al., 2012; Chhibber et al., 2013; Mendes et al., 2013; Seth et al., 2013; Drilling et al., 2014; Gutiérrez et al., 2015). The two bacteriophages selected for this analysis, phiIPLA-RODI and phiIPLA-C1C, have been isolated from sewage treatment plant samples and exhibit antibiofilm potential (Gutiérrez et al., 2015). In order to evaluate the factors affecting penetration into and propagation inside the biofilm, several strains were selected to represent varying biofilm-forming abilities and phage susceptibility profiles. Once established the bacterial strains and phages used to set up the model, penetration of phage suspensions through different monospecies biofilms was tested. These phage suspensions were prepared to represent a high (∼10^9^ PFU/ml) or low (∼10^6^ PFU/ml) viral concentration. The results from these experiments confirmed that the phage could effectively diffuse through all the different biofilms tested at both concentrations, although the phage titer determined in the flow-through varied for different bacterial strains. Analysis of these changes suggests that the phenotype that has a greater effect on the net phage titer after diffusion through the biofilm is phage susceptibility of the strain forming the biofilm. Indeed, a correlation between susceptibility and phage titer was observed for phiIPLA-RODI at both concentrations tested and low concentrations of phiIPLA-C1C. Perhaps, this is partly due to the ability of the phage, especially at high doses, to kill susceptible cells and potentially disrupt the biofilm structure or its ability to cross the biofilm by propagating “from cell to cell” instead of having to move through the extracellular matrix. However, in some cases, the best correlation was obtained when both susceptibility and biofilm formation were taken into account, which indicates that the strength of the biofilm may also affect phage penetration across the structure. Nonetheless, the complex and varied architecture of bacterial biofilms, as observed in this study, are also likely to play a role in the ability of the bacteriophages to move across the biofilm, with the virus potentially taking advantage of thinner areas, especially in thick highly structured biofilms. Indeed, this would be an interesting topic to examine further in subsequent studies.

The subsequent studies performed with phage phiIPLA-RODI provided a closer look at the interaction between this phage and different bacterial biofilms. Thus, when mixed-strain biofilms were challenged with a low concentration of phiIPLA-RODI, the results were quite varied and, sometimes, unexpected. For example, *L. plantarum* 55-1 and *S. epidermidis* F12 exhibited a similar behavior when forming monospecies biofilms, reducing the phage titer by approximately 2 log units. However, biofilms formed by different *S. aureus* strains with *L. plantarum* 55-1 always led to a lower phage titer in the flow-through than in the control. In contrast, mixed biofilms of *S. aureus* strains with *S. epidermidis* F12 did not decrease phage titer. This suggested that the mechanisms by which these two species decrease the number of phage particles in the flow-through may be different and display different interactions with the phage propagation/phage inactivation dynamics of *S. aureus* strains. This hypothesis seems even more likely in view of the fact that filtered supernatants of *L. plantarum* appear to inactivate phiIPLA-RODI, whereas those of *S. epidermidis* F12 reduced but did not eliminate the phage below the detection level. More research is, nevertheless, necessary to explore the mechanism that leads to a decrease in the number of viable phage particles in the latter strain. Interestingly, a previous study had shown that phiIPLA-RODI propagation in biofilms formed by *S. aureus* IPLA16 with *L. plantarum* 55-1 was lower than in biofilms formed with *Lactobacillus pentosus* (González et al., 2017). This may also be a consequence of phage inactivation in the presence of *L. plantarum* but not *L. pentosus*. Additionally, analysis of the viable phage particles trapped in the extracellular matrix of different biofilms showed interesting results. For instance, strains *S. epidermidis* F12 and *L. plantarum* 55-1 did not appear to have retained a high proportion of the viral particles within the matrix. This suggests that the low titer in the flow-through may be due to inactivation of the phage in the biofilms formed by these two strains. However, further research is necessary to establish the specific mechanism involved in this inactivation, which may involve enzymatic degradation or irreversible binding of the phage to cell debris or matrix components. Another interesting detail is the fact that the biofilm matrix of *S. aureus* IPLA 15 and *S. aureus* V329 exhibit a higher phage titer than that expected from the levels observed in the flow-through. As mentioned above, this may be related to differences in biofilm architecture or composition of the extracellular matrix. Of note, *S. aureus* V329 is the only strain analyzed that has a protein-based extracellular matrix. With regard to *S. aureus* IPLA15, a previous study suggested that exposure to subinhibitory levels of phage phiIPLA-RODI led to accumulation of extracellular DNA in the biofilm (Fernández et al., 2017).

**FIGURE 5:**
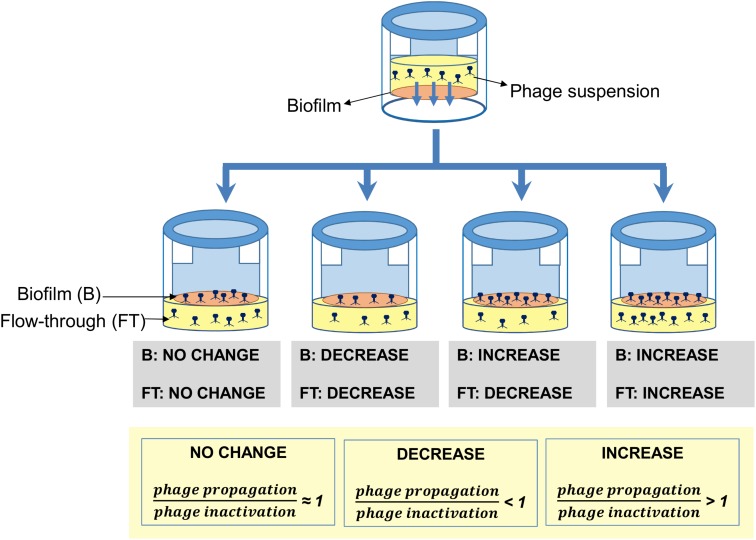
Schematic representation of the different outcomes observed when preformed biofilms formed by different strains were treated with phage phiIPLA-RODI. In the biofilm, these results represent the overall balance between phage propagation and phage inactivation due to enzymatic activity or irreversible binding to the extracellular matrix or cell debris. In the flow-through, besides the aforementioned factors, net diffusion across the biofilm also contributes to the final outcome.

Taking into account all these results, it can be concluded that the outcome of phage penetration across biofilms is the result of the net balance between phage propagation and phage inactivation in the biofilm, as well as phage penetration and diffusion into the biofilm (**Figure 5**). These processes will largely depend on the bacterial strains forming the biofilm on the basis of different properties. For instance, phage susceptibility of the strains in the biofilm will determine the propagation rate of the phage inside the biofilm. Conversely, entrapment of the phage particles may depend on the composition of the biofilm matrix or the bacterial cell surface. Finally, production of phage-inactivating enzymes and/or lowering of the pH by a bacterial strain in the community may have a deleterious effect on the phage population that cannot be overcome by phage propagation.

In conclusion, this study describes a new technique for the analysis of phage-bacteria interactions within biofilms and shows an example of its potential. Indeed, application of this protocol to staphylophages has revealed interesting aspects of phage-host interplay in biofilms that will be useful for the development of phage-based products aimed at the elimination of staphylococcal biofilms. In general, it seems that the two phages tested here can diffuse through biofilms formed by strains with different degrees of susceptibility and biofilm-forming abilities. This highlights the viability of phage-based biocontrol of bacterial biofilms. However, net diffusion depends on several factors, including phage concentration, biofilm-forming capability, susceptibility to the phage, phage inactivation and, potentially, changes in biofilm structure as a response to phage predation. As a result, all these conditions need to be evaluated to design the adequate composition of a phage-based product, especially in terms of phage concentration and addition of compounds that can inhibit undesired effects like phage inactivation in the biofilms. Furthermore, this technique provides a new tool for decrypting the complex dynamics of phage infection inside sessile microbial communities. Given that biofilms are the most widespread mode of bacterial growth in nature and that bacteriophages are very numerous, these interactions are bound to have an impact on the structure and physiology of microbial communities in natural environments.

## Author Contributions

SG, LF, AR, and PG conceived and designed the experiments and analyzed the data. SG, LF, and AC performed the experiments. SG, LF, DG, AR, and PG wrote the paper.

## Conflict of Interest Statement

The authors declare that the research was conducted in the absence of any commercial or financial relationships that could be construed as a potential conflict of interest.
