# Supplementary material for: Analysis of Different Parameters Affecting Diffusion, Propagation and Survival of Staphylophages in Bacterial Biofilms
Source: Front Microbiol. 2018 Sep 28;9:2348. doi: 10.3389/fmicb.2018.02348 (PMC6172340; doi:10.3389/fmicb.2018.02348)

Supplementary Material

Analysis of Different Parameters Affecting Diffusion, Propagation and Survival of Staphylophages in Bacterial Biofilms

Silvia González[1], Lucía Fernández[1]*, Diana Gutiérrez[1], Ana Belén Campelo[1], Ana Rodríguez[1] and Pilar García[1]

[1] Instituto de Productos Lácteos de Asturias (IPLA-CSIC). Paseo Río Linares s/n, 33300, Villaviciosa, Asturias, Spain.

*** Correspondence:**
Lucía Fernández
lucia.fernandez@ipla.csic.es

FIGURE S1 | Biofilm formation of different bacterial strains on polycarbonate membranes after 24 hours of incubation at 37ºC. A) The depicted values correspond to the average and standard deviation of at least three independent repeats. Sa, *S. aureus*; Se, *S. epidermidis* and Lp, *L. plantarum*. Strong ($A_{595}>2$), intermediate ($1<A_{595}<2$) and weak ($A_{595}<1$) biofilm formers are represented in black, grey and white bars, respectively. The P-values shown in the table below the histogram were obtained comparing biofilm formation of the different strains. P-values < 0.05 were considered significant (light grey cells). B) Linear regression analysis between average values of biofilm formation on polycarbonate membranes (quantified as A595 of crystal violet stained biofilms) and biofilm thickness (in µm) as determined by confocal microscopy of biofilms formed in glass and stained with SYTO 9 are also shown.

A

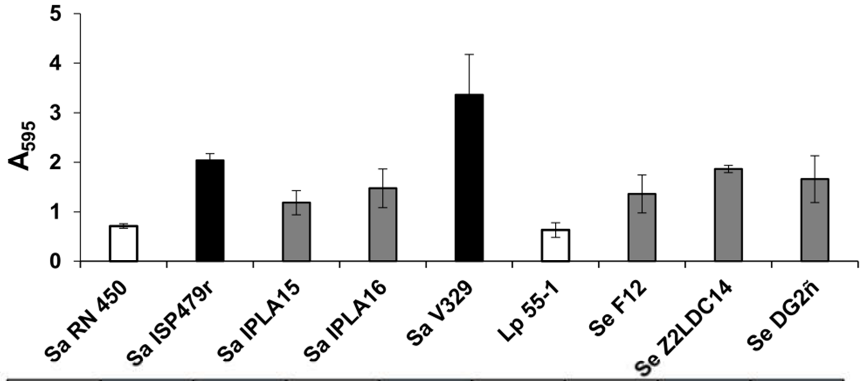

**Sa RN450**		**0.002**	**0.028**	0.076	**0.030**	0.447	0.098	**0.000**	**0.028**
**Sa ISP479r**	**0.002**		**0.002**	0.116	0.103	**0.000**	0.078	0.166	0.218
**Sa IPLA15**	**0.028**	**0.002**		0.167	**0.019**	**0.007**	0.267	**0.004**	0.071
**Sa IPLA16**	0.076	0.116	0.167		**0.040**	0.050	0.733	0.223	0.594
**Sa V329**	**0.030**	0.103	**0.019**	**0.040**		**0.029**	**0.035**	0.086	**0.049**
**Lp 55-1**	0.447	**0.000**	**0.007**	0.050	**0.029**		0.067	**0.001**	**0.018**
**Se F12**	0.098	0.078	0.267	0.733	**0.035**	0.067		0.073	0.198
**Se Z2LDC14**	**0.000**	0.166	**0.004**	0.223	0.086	**0.001**	0.073		0.231
**Se DG2ñ**	**0.028**	0.218	0.071	0.594	**0.049**	**0.018**	0.198	0.231	

B

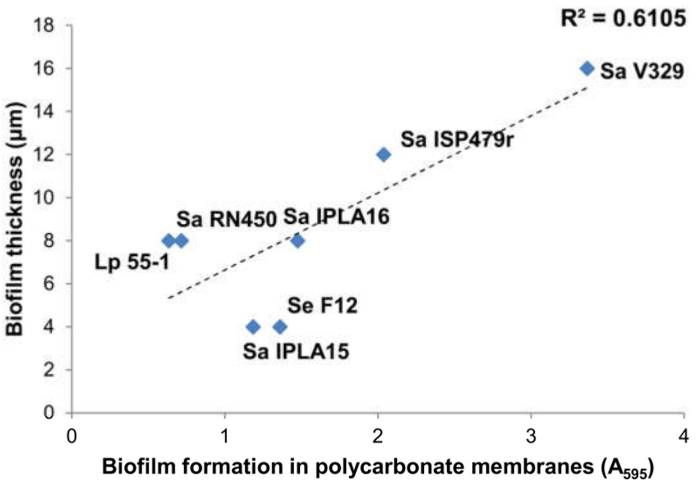

FIGURE S2 | Confocal microscopy analysis of biofilms formed by different *S. aureus* strains. Biofilms were allowed to form on glass-bottomed slides for 24 h at 37º C and then washed with PBS and stained with SYTO 9 prior to observation with a confocal laser scanning microscope. The two images for each strain respectively show a 2D view of the biofilm surface (top) and a lateral view of the biofilm (bottom).

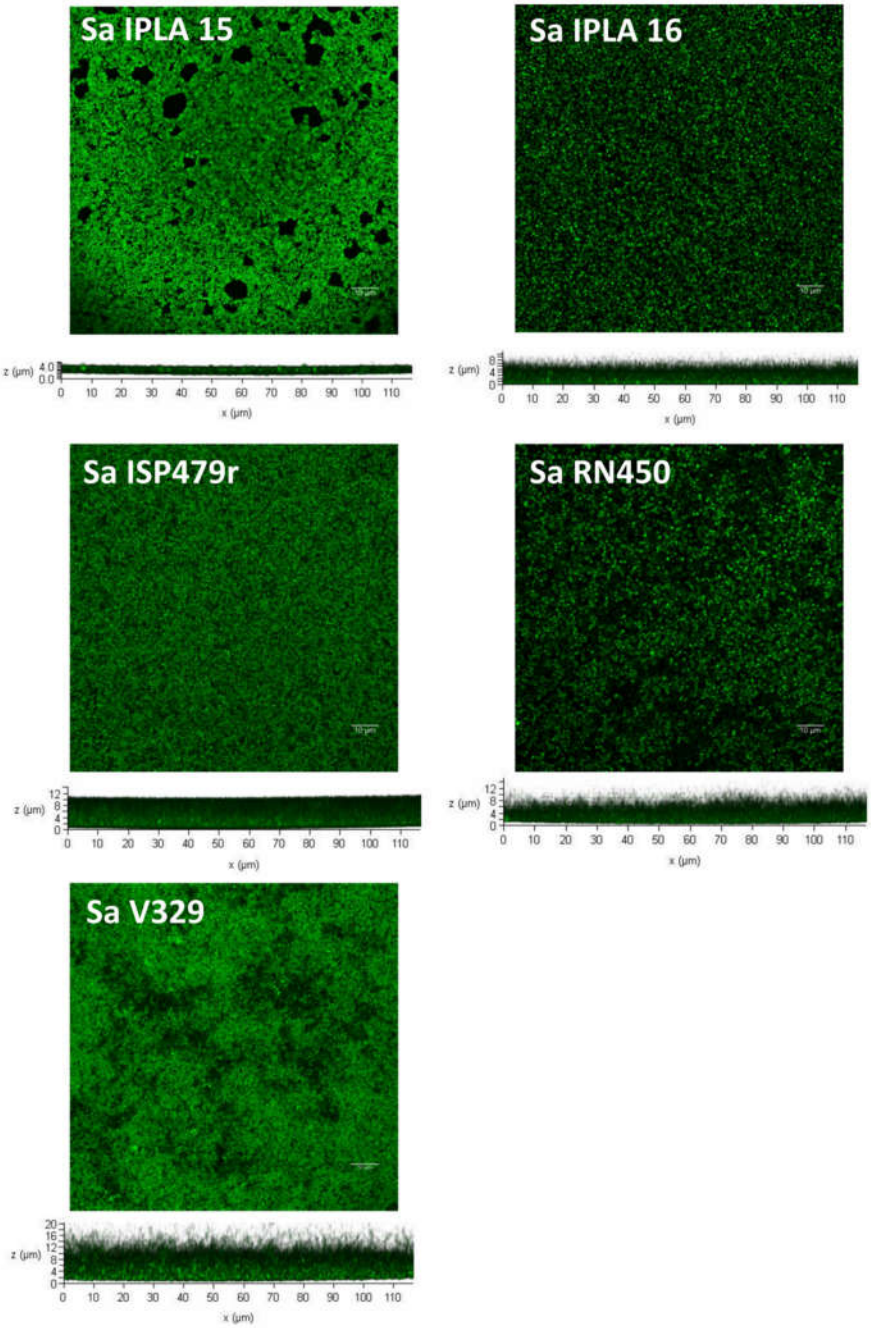

FIGURE S3 | Confocal microscopy analysis of biofilms formed by different *S. epidermidis* and *L. plantarum* strains. Biofilms were allowed to form on glass-bottomed slides for 24 h at 37º C and then washed with PBS and stained with SYTO 9 prior to observation with a confocal laser scanning microscope. The two images for each strain respectively show a 2D view of the biofilm surface (top) and a lateral view of the biofilm (bottom).

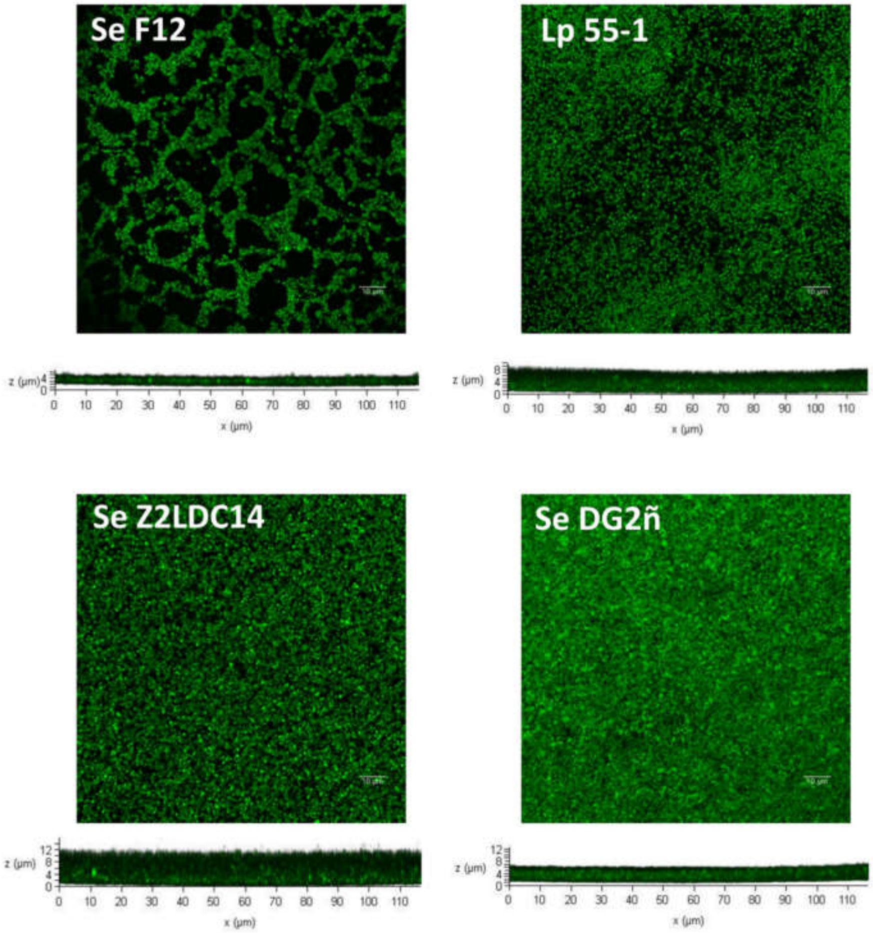

FIGURE S4 | Linear regression analysis of the relationship between biofilm formation, phage susceptibility or a combination of both and phage titer in the flow-through of biofilms formed by different bacterial strains treated with phiIPLA-RODI. The graphs on the left and right correspond to treatment with a high (10^9 PFU/ml) and low (10^6 PFU/ml) phage concentration, respectively. The equation of the trend line is shown in the upper left corner of each chart. The goodness of fit of the trend line estimated as coefficient of determination (R^2) is shown in the upper right corner of each chart.

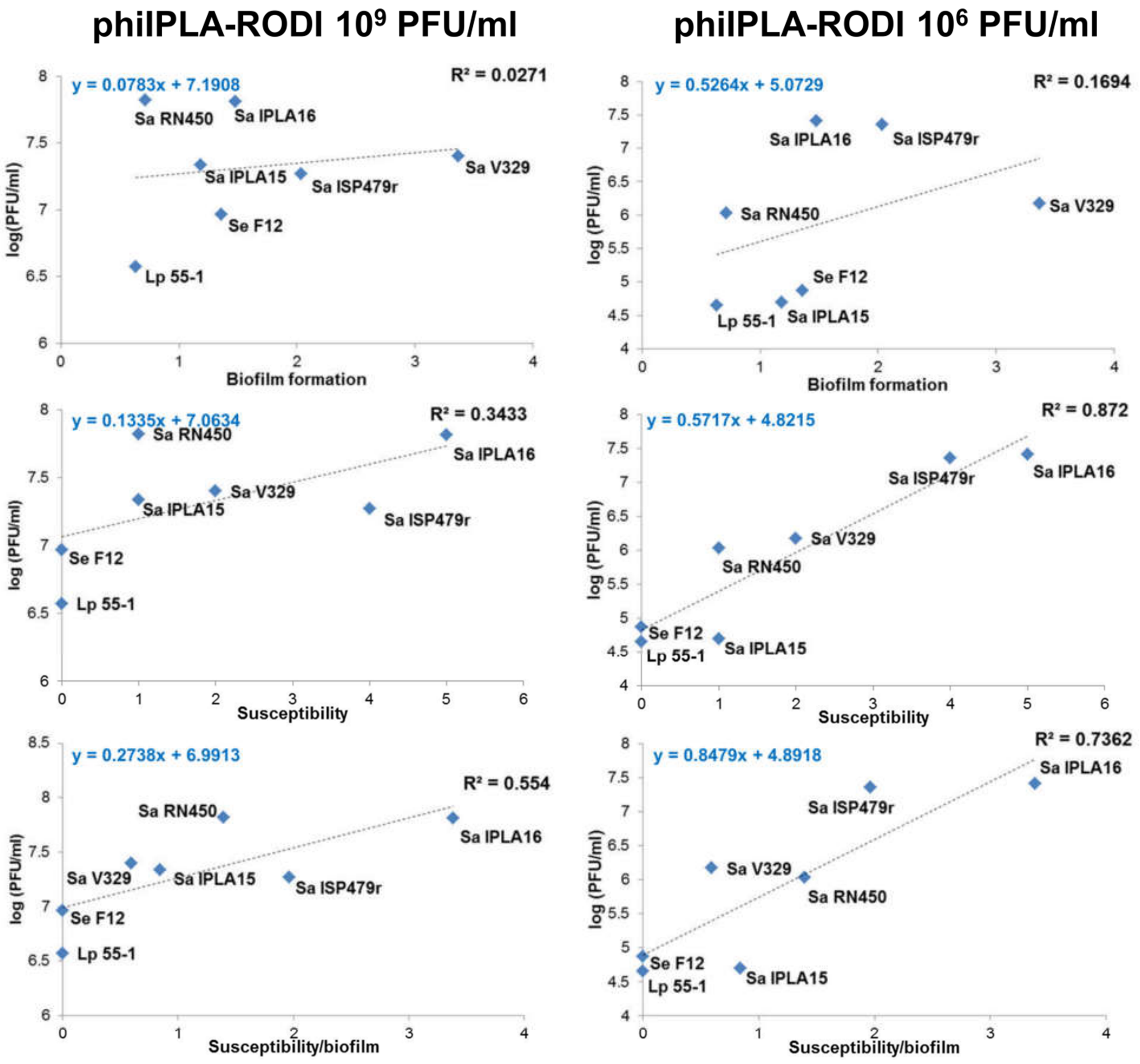

FIGURE S5 | Linear regression analysis of the relationship between biofilm formation, phage susceptibility or a combination of both and phage titer in the flow-through of biofilms formed by different bacterial strains treated with phiIPLA-C1C. The graphs on the left and right correspond to treatment with a high (10^9 PFU/ml) and low (10^6 PFU/ml) phage concentration, respectively. The equation of the trend line is shown in the upper left corner of each chart. The goodness of fit of the trend line estimated as coefficient of determination (R^2) is shown in the upper right corner of each chart.

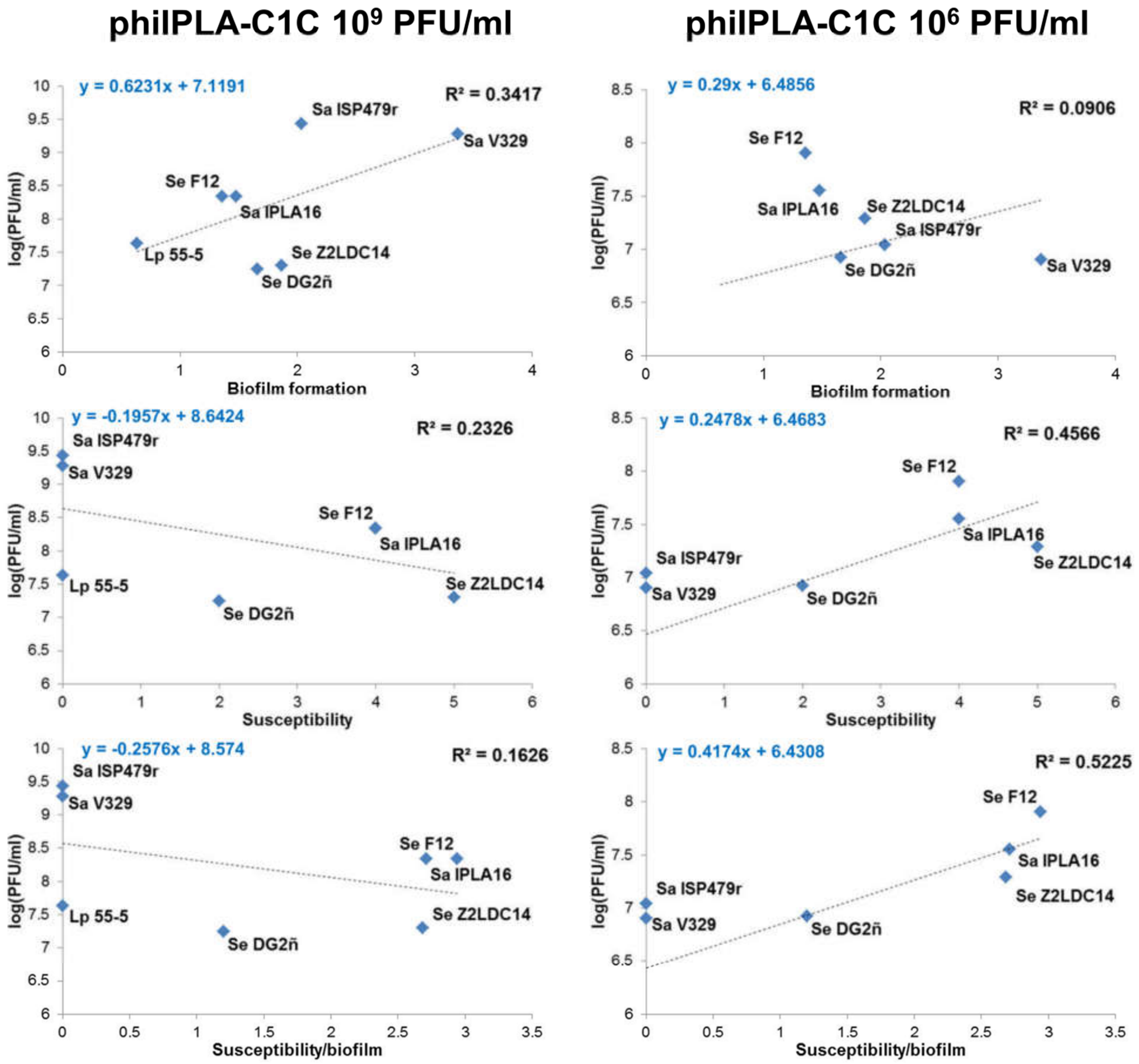

FIGURE S6 | Linear regression analysis of the relationship between number of phage particles entrapped in the matrix and phage titer in the flow-through of biofilms formed by different bacterial strains treated with 10^6 PFU/ml of phiIPLA-RODI. The graph on the left corresponds to the analysis carried out including all strains, while the graph on the right represents the analysis performed excluding strain *S. aureus* IPLA 15. The equation of the trend line is shown in the upper left corner of each chart. The goodness of fit of the trend line estimated as coefficient of determination (R^2) is shown in the upper right corner of each chart.

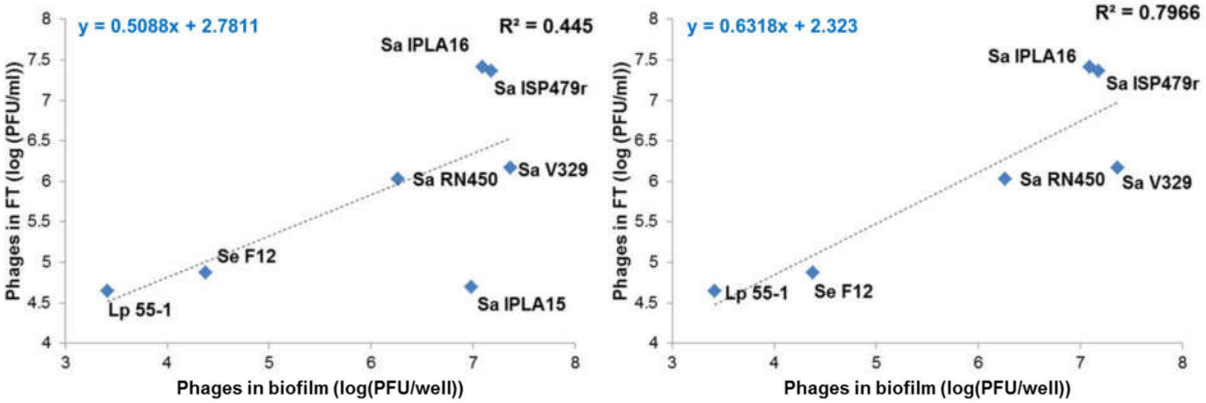

Supplement: Supplementary file 1 [file Data_Sheet_1.pdf]
